# The effect of alkyl termination on the optical and electronic properties of silicon nanoparticles†

**DOI:** 10.1039/d5ra03272e

**Published:** 2025-06-17

**Authors:** Eimear Madden, Martijn A. Zwijnenburg

**Affiliations:** a Department of Chemistry, University College London 20 Gordon Street London WC1H 0AJ UK m.zwijnenburg@ucl.ac.uk

## Abstract

In this study, we use a combination of (time-dependent) density functional theory and many-body perturbation theory methods to study the impact of alkyl termination on the optical and electronic properties of silicon nanoparticles (SiNPs), as well as the effect of increasing particle size. A comparative study of hydrogen and methyl-terminated SiNPs reveals that replacing hydrogen atoms with methyl groups results in a reduction of the fundamental gap, optical gap, and exciton binding energy. The effect of replacing hydrogen by methyl diminishes with the increasing size of the silicon core of the particles, which can be attributed to the decreasing surface-to-volume ratio. Larger hydrogen-terminated SiNPs, therefore, serve as increasingly accurate models for alkyl-terminated SiNPs. The size of the lowest energy excited-state (exciton) increases when replacing (more of the) hydrogen atoms with methyl groups for a given silicon core size, suggesting that the exciton delocalises onto the methyl groups. Analysis of the relevant natural transition orbitals confirms that both the excited electron and hole components of the exciton partially delocalise on to the methyl groups, with increased delocalisation in the case of the excited electron. The reduced fundamental and optical gaps and exciton binding energy in methyl-terminated SiNPs, and probably by extension alkyl terminated SiNPS in general, are likely due to the electron-donating nature of methyl groups combined with exciton delocalisation.

## Abstract 2

Many-body perturbation theory and (time-dependent) density functional theory are used to study the effect of alkyl termination on the optical and electronic properties of silicon nanoparticles.

## Graphical abstract

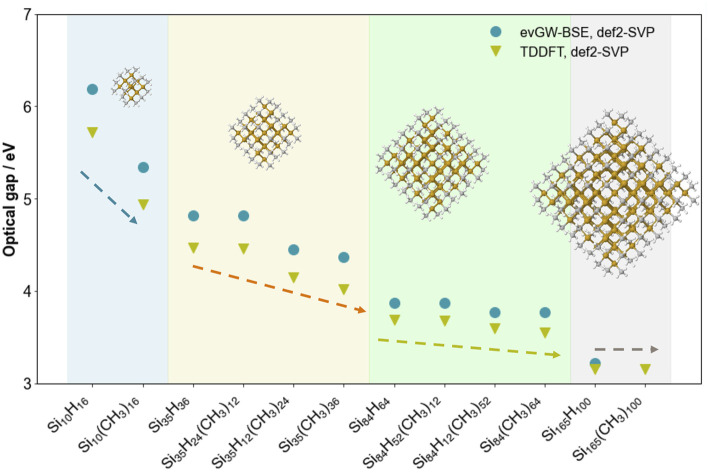

## Introduction

Silicon nanoparticles (SiNPs), either embedded in a solid matrix or as colloidal particles, find use in a range of applications. They are used, for example, in light emitting diodes, lasers, solar-cells, data storage, as well as in bioimaging and sensing.^1–3^ These applications exploit the fact that the optical and electronic properties of such nanoparticles can be tuned by changing the size (distribution) of the particles and are different from those of bulk silicon. The optical gap, corresponding to the onset of light absorption, and the fluorescence maximum both shift to higher energies, and thus shorter wavelengths, with decreasing particle size, while the exciton lifetime decreases. As such, SiNPs are also the structurally and chemically simplest realisation of the concept of semiconductor nanoparticles, often referred to as quantum dots colloquially.

The structure of these SiNPs consists of two parts: (i) a silicon core in which silicon has the same diamond structure as bulk silicon and (ii) a layer of capping groups that cap or terminate the otherwise undercoordinated atoms on the surface of the silicon core.^4^ Because of the covalent bonding in silicon, the absence of such capping groups would result in dangling bonds and a large reconstruction of the surface of the silicon core to eliminate these dangling bonds.

The simplest capping group is a hydrogen atom. While silicon SiNPs can be capped with hydrogen atoms, many silicon nanoparticles, especially colloidal particles synthesized for sensing and imaging applications, are capped with more complicated organic capping groups. Such organic groups are generally specifically introduced during the synthesis of the silicon nanoparticles, for example, so that the synthesised nanoparticles have a special affinity to a biomolecule they will be used to sense^2^ or to improve the stability of the SiNP against oxidation, as hydrogen-terminated SiNPs are prone to oxidise.^5^

Most computational work in the literature focusses on hydrogen capped SiNPs nanoparticles because of their higher symmetry and simpler structures.^6–21^ However, it is important to understand the effect of larger capping groups than a hydrogen atom on the optical and electronic properties of the SiNPs, and the extent to which the electronically and optically excited states of the particles involve these capping groups.

Previous computational work in this area includes a study by Reboredo and Galli,^22^ who showed that the energy difference between the highest occupied and lowest unoccupied Kohn–Sham orbitals for a SiNP, which they use as a proxy for the optical gap of their particles, does not significantly change when replacing hydrogen atoms by alkyl chains. The highest occupied and lowest unoccupied Kohn–Sham orbitals themselves, which can be thought of as a proxy for the ionisation potential and electron affinity of the particle, show a more dramatic change and shift to less negative values. The same group later revisited the absorption spectrum of these hydrogen and alkyl terminated SiNPs with time-dependent density functional theory (TD-DFT),^11^ which allows one to predict the optical gap directly, and similarly report a negligible change in the onset of light absorption, even if the spectrum above the optical gap is predicted to change. Li and co-workers studied the absorption spectrum of SiNPs in which part of the capping hydrogen atom have been replaced by propionic acid groups using time-dependent tight-binding density functional theory and again predict only a modest shift in the optical gap.^23^ Finally, Dohnalová and co-workers studied the effect of replacing hydrogen atoms by organic capping groups on the exciton lifetime and radiative rates for SiNPs using separately tight-binding calculations,^24^ and density functional theory (DFT)^25^ and predicted that the exciton lifetime reduces and radiative rates increase when replacing hydrogen atoms, as well as again that the gap between occupied and unoccupied Kohn–Sham states does not significantly change.

**Fig. 1:**
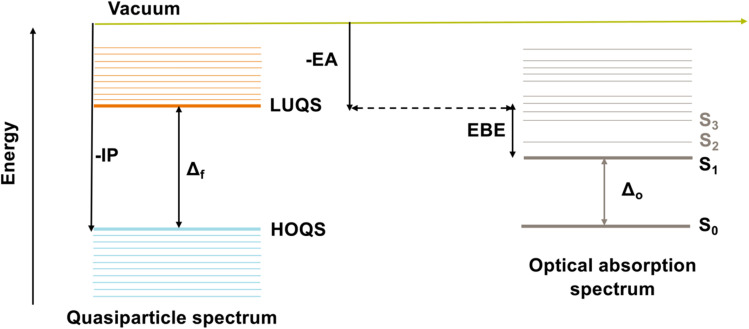
A schematic illustrating the quasiparticle and optical spectra, highlighting the definitions of the ionisation potential (IP), electron affinity (EA), fundamental gap (Δ*f*), optical gap (Δ*o*) and exciton binding energy (EBE), which is the difference between Δ*f* and Δ*o*. In this context, HOQS represents the highest occupied quasiparticle state, LUQS the lowest unoccupied quasiparticle state, *S*_0_ the electronic ground state, and *S*_1_ the first singlet electronic excited state.

**Fig. 2:**
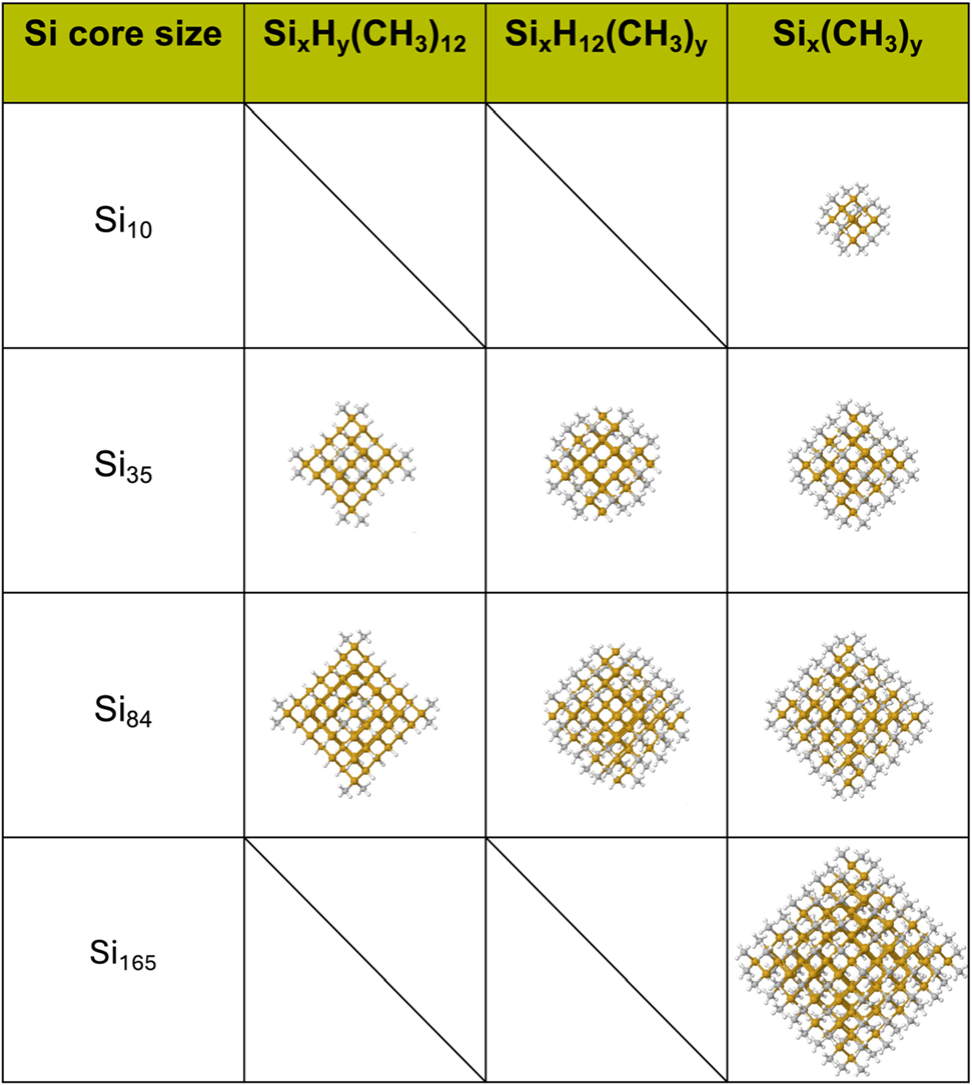
Structures of the partially and fully methyl-terminated SiNPs, optimised in *C*_1_ using the B3LYP functional and the def2-SVP basis-set.

Here, we use a combination of (TD-)DFT, and many-body perturbation theory, GW and GW combined with solving the Bethe–Salpeter equation (BSE), to understand the changes in the optical and electronic properties of SiNPs when replacing the capping hydrogen atoms with organic groups. We build on our previous work on hydrogen terminated SiNPs, in which we also used the same combination of (TD-)DFT and GW(-BSE).^20^ Use of GW-BSE allows us to calculate internally consistent optical and fundamental gap values, as well as exciton binding energy values, which would be impossible with (TD-)DFT. The exciton binding energy (EBE) is a measure of how much the exciton (the excited electron–hole pair) corresponding to the lowest singlet excited state, the optical gap, is stabilised by the electrostatic interaction between excited electron and hole. The EBE is defined as the difference between the particle's fundamental and optical gaps (see Fig. 1). TD-DFT on the other hand allows us to calculate properties for particles too big for GW-BSE or spectra with more excited-states than tractable with GW-BSE. We consider three particles with cores containing 10, 35 and 84 silicon atoms, respectively, and consider particles where we have replaced all hydrogen atoms by methyl groups and particles where only the corner hydrogen atoms or only the hydrogen atoms on the faces of the particles have been replaced with methyl groups, as shown in Fig. 2.

### Methodology

In this paper we build upon our previous work on hydrogen-terminated SiNPs. The starting structures for the methyl-terminated SiNPs were obtained by replacing the hydrogen atoms in the optimised hydrogen-terminated SiNP structures by methyl groups. The starting structures for the hydrogen-terminated SiNPs in turn were obtained in our earlier study.^20^ Here the Nanocut code was used,^26^ which cut the silicon cores of the nanoparticles out of a supercell of bulk diamond silicon, based on structural data obtained from the Materials Project,^27^ after which we manually terminated any undercoordinated atoms on the surface with hydrogen atoms.

The structures of the methyl-terminated SiNPs were optimised using DFT calculations, employing the B3LYP hybrid density functional,^28–30^ in combination with the D3 dispersion correction by Grimme and co-workers,^31^ along with Becke-Johnson damping, and the def2-SVP or def2-TZVP basis sets.^32^ All calculations on the methyl-terminated SiNPs were performed in the absence of any enforced symmetry (*C*_1_). The hydrogen-terminated SiNPs have *T*_d_ symmetry, but the methyl groups reduce this symmetry to *C*_1_ for all particles other than Si_10_(CH_3_)_16_, even if the silicon core remains approximately *T*_d_. Harmonic frequency calculations were performed, where feasible, to ensure that the optimised structures correspond to minima with only positive and no imaginary frequencies.

Subsequently, the quasiparticle spectrum, specifically the highest occupied and lowest unoccupied quasiparticle states, or the ionisation potential and electron affinity, and the gap between them, the fundamental gap, of the DFT-optimised particles were predicted using GW. Different GW variants exist: single-shot *G*_0_*W*_0_, eigenvalue-only self-consistent GW (evGW), and quasiparticle self-consistent GW (qsGW) calculations,^33–36^ where here we will focus on evGW calculations. These evGW calculations utilised the B3LYP orbitals as starting points and again used the def2-SVP or def2-TZVP basis sets. The results of these evGW calculations served as inputs for solving the Bethe–Salpeter equation (BSE) to obtain vertical excitation energies, oscillator strength values, and ultimately the nanoparticles' optical gap values.^37^

For *G*_0_*W*_0_, and by extension *G*_0_*W*_0_/BSE, the predicted properties will show a dependency on the functional used in the underlying DFT calculation. evGW and qsGW reduce and practically eliminate, respectively, this starting-point dependency by iterating the eigenvalues or the underlying ground state, respectively, until self-consistency is achieved.^34^ Moreover, in the case of finite-sized systems as studied here, the results of evGW and qsGW and solving the Bethe–Salpeter equation agree well with coupled-cluster benchmarks, as explicitly shown for singlet excitation energies for organic molecules,^35^ and yield excitation energies there that are clearly superior to those obtained from *G*_0_*W*_0_-BSE. The reason we focus here on evGW instead of qsGW is that the implementation of the former in Turbomole, where only the highest occupied and lowest unoccupied quasiparticle states are calculated explicitly using GW while the remainder of the quasiparticle states are the Kohn–Sham states, or more strictly the generalised Kohn–Sham states as we use a hybrid functional, shifted accordingly, is computationally much more efficient.

Additionally, time-dependent density functional theory (TD-DFT) calculations were carried out on the DFT-optimised SiNPs, again using the same B3LYP functional and def2-SVP basis set to predict the particle's absorption spectrum. These TD-DFT calculations, in contrast to the BSE calculations, make the Tamm–Dancoff approximation to avoid TD-DFT stability issues.^38^ We also compare the GW fundamental gap value and IP and EA to the DFT Kohn–Sham, or more strictly generalised, Kohn–Sham, as we use a hybrid density functional, gap and energies of the highest occupied and unoccupied orbitals.

B3LYP was used throughout as in previous work use of this functional was found to yield good results for hydrogen-terminated SiNPs. Specifically, we found that the results of qsGW(-BSE) and evGW(-BSE) for these particles agreed well, suggesting a minimal residual starting point dependence when using B3LYP. Similarly, TD-DFT calculations with the B3LYP functional yielded excitation energies that were similar to those obtained using evGW/BSE and qsGW/BSE.

Throughout all these calculations, version 7.5 of the Turbomole code was employed in combination with a tight integration grid (m5), stringent SCF convergence criteria (denconv. set at 1 × 10^−7^), and the resolution of identity (RI-J) approximation, which accelerated the computation of the Coulomb integrals.^39–41^ Additionally, the RI-K approximation was applied in all BSE calculations.

The bandlike nature of the frontier orbitals and natural transition orbitals (NTOs) was studied by carrying out a discrete Fourier transform on the orbitals or NTOs.^42^ This Fourier transform was performed using an inhouse Python code based around routines from the *numpy* library and with a cube file of the orbitals/NTOS as an input.^43^ The Fourier transform was plotted as a function of *k*_*x*_ and *k*_*y*_ defined as:

1 *k*_*x*/*y*_ = 2π/*l*_*x*/*y*_

with the information along the *z*-direction projected on the *xy* plane by summing up the contributions along the *z*-axis for each *xy* value.

## Results

### Fundamental and optical gap

**Fig. 3:**
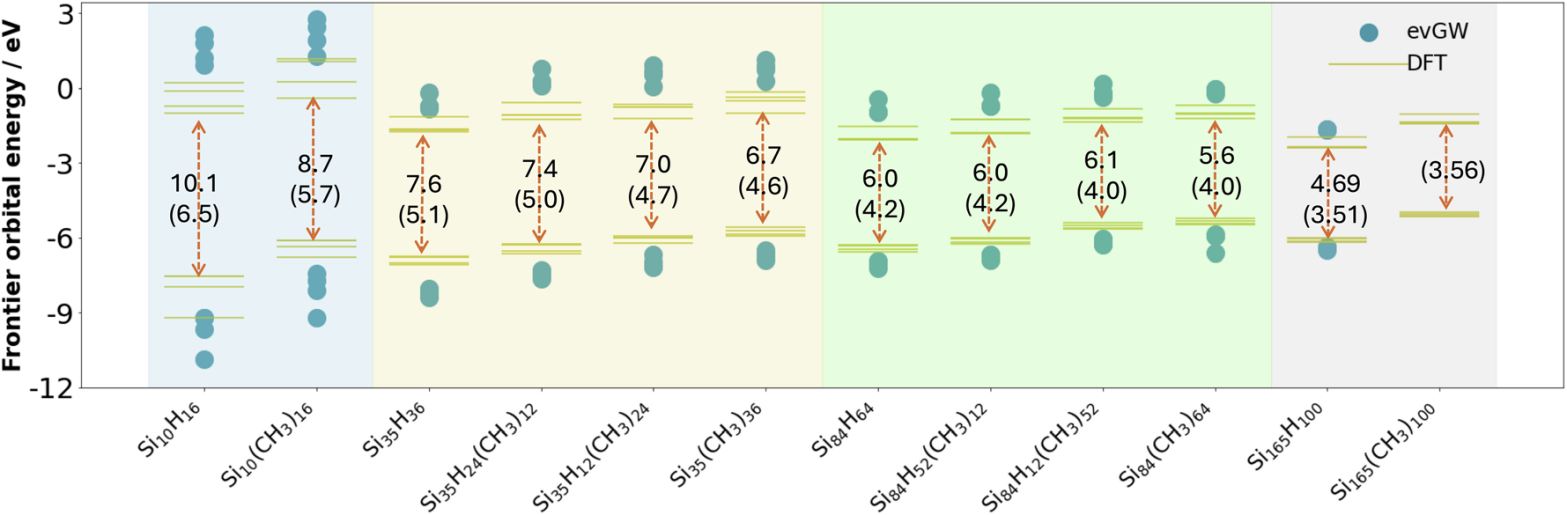
Predicted quasiparticle spectrum for the hydrogen and methyl-terminated SiNPs studied, showing the four highest occupied and the four lowest unoccupied Kohn–Sham orbitals/quasiparticle states obtained from DFT and evGW, respectively. For each particle the evGW predicted fundamental gap values and the DFT Kohn–Sham gap are also shown, the latter in between parentheses. All results obtained with the def2-SVP basis-set. To aid visualisation, the results are divided into coloured sections based on the number of silicon atoms in the nanoparticle (blue, yellow, green and grey for 10, 35, 84, and 165 silicon atoms, respectively).

**Fig. 4:**
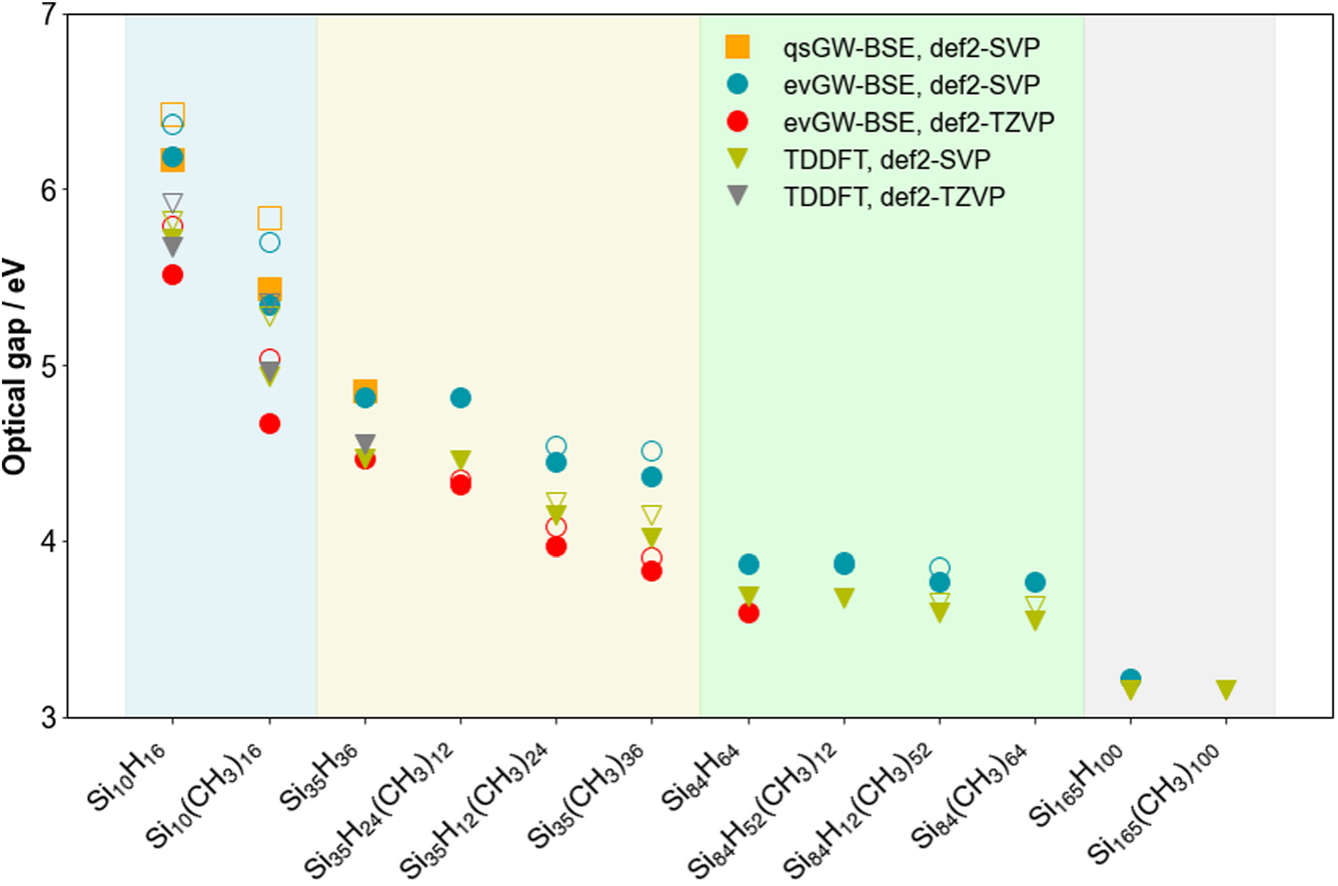
Plot of the optical gap values predicted using TD-DFT, evGW-BSE and qsGW-BSE for the different SiNPs considered. In instances where the lowest optical excitation has a low enough oscillator strength to be considered dark (∼10^−7^), the lowest lying excitation plus the first excitation which has a high enough oscillator strength to be considered bright (∼10^−3^, open symbol), is plotted. All results obtained with def2-SVP. To aid visualisation, the results are divided into coloured sections based on the number of silicon atoms in the nanoparticle (blue, yellow, green and grey for 10, 35, 84 and 165 silicon atoms, respectively).

Fig. 3 and 4 show how the fundamental and optical gap values of the SiNPs change when replacing hydrogen capping atoms with methyl groups and/or increasing the size of the silicon core. In both cases introducing methyl groups results in a reduction of the gap, where this reduction is most pronounced when all the hydrogen atoms are replaced by methyl groups. The effect of introducing methyl groups is also the most pronounced for the smallest particle, probably because this is effectively all surface, and gets progressively smaller with increasing particle size. The predicted magnitude of the change in gaps upon introduction of methyl groups is in line with previous work,^21–23,25^ in the sense that it is small and could easily be interpreted as no change when only studying larger particles. The predicted optical gaps are also consistent with the lowest absorption edge (320–280 nm/3.87–4.4 eV) reported experimentally for 1–2 nm alkyl terminated silicon nanoparticles.^44,45^ Switching our focus to the effect of particle size; the fundamental and optical gap of the (partly) methyl-terminated particles display a similar trend as their fully hydrogen-terminated counterparts, where both gaps increase with decreasing particle size.

Focussing in on the positions of the highest occupied and lowest unoccupied quasiparticle state relative to vacuum, here introduction of methyl groups results in an upward shift of both, *i.e.* they both become shallower. The ionisation potential and electron affinity of SiNPs hence both becomes less negative when replacing hydrogen atoms by methyl groups, where generally the shift is larger for the ionisation potential than the electron affinity. Again, this effect is most pronounced for the smaller particles and qualitatively in line with previous predictions in the literature for the change in Kohn–Sham orbital energies upon replacing hydrogen atoms by alkyl groups.^22^

For Si_35_H_36_ and Si_84_H_64_, we found in our previous work that the excited state responsible for the optical gap was bright in both cases and symmetry allowed, even if for Si_84_H_64_ this bright state is effectively degenerate with a non-bright symmetry forbidden excitation. Concentrating on the evGW-BSE results, we find that the lowest excited state for Si_35_H_24_(CH_3_)_12_ is also bright, while for Si_84_H_52_(CH_3_)_12_ we find, just as for Si_84_H_64,_ that the lowest two excited states are an effectively degenerate pair of a bright and non-bright state. However, for Si_35_H_12_(CH_3_)_24_, Si_35_(CH_3_)_36_, Si_84_H_12_(CH_3_)_52_ an Si_84_(CH_3_)_64_, the optical gap corresponds to a clearly non-bright state, probably derived from what would be a symmetry forbidden state for the hydrogen-terminated SiNP. The first bright state for these particles lies ∼0.1 eV higher in energy and the gap between the non-bright and lowest bright state appears to decrease with particle size.

Just as we previously observed for the hydrogen-terminated SiNPs, evGW-BSE and (TD-)DFT give comparable results and predict very similar trends. The main difference is that the DFT Kohn–Sham gap underestimates the fundamental gap relative to the values predicted by evGW-BSE. Linked to that, the highest occupied and lowest unoccupied Kohn–Sham states are also consistently more shallow and less shallow, respectively, than their evGW counterparts. For Si_165_(CH_3_)_100_, where only TD-DFT calculations are tractable, these calculations show that just as for the smaller fully methyl terminated SiNPs the lowest excited-state is non-bright but that the first bright state is essentially degenerate (difference <0.05 eV), as well as that the optical gap of the hydrogen and methyl-terminated particles is essentially the same. Finally, just as the hydrogen-terminated SiNPs, qsGW(-BSE), here only tractable for Si_10_(CH_3_)_16_, gives very similar results to those obtained with evGW(-BSE) (see Fig. 4, Tables S1, S2, S4 and S5†).

### Excitonic character

**Fig. 5:**
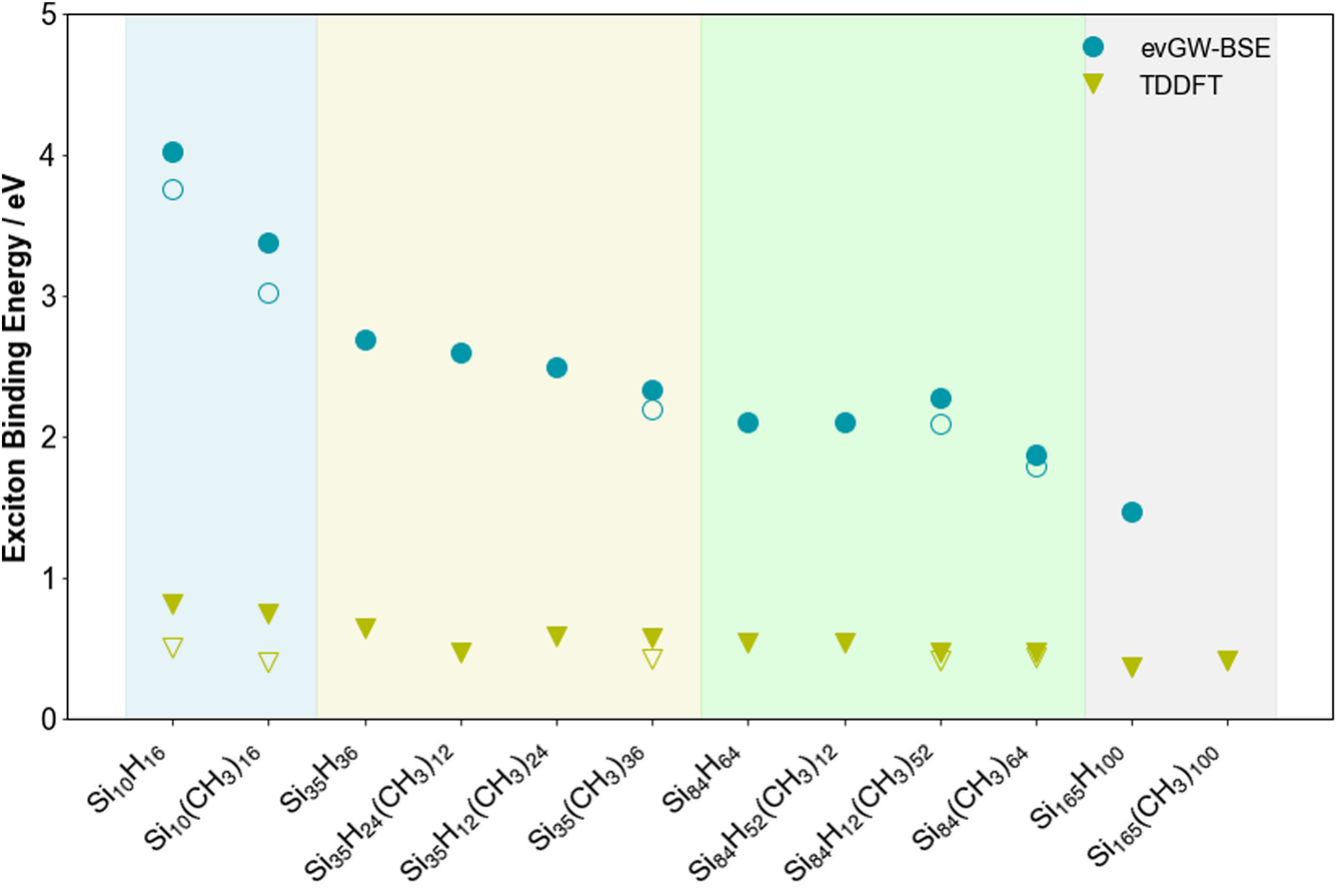
Predicted exciton binding energy values for the lowest excited states of the hydrogen and methyl-terminated SiNPs. Additionally, in the instances where the lowest lying excitation is dark, the exciton binding energy of the lowest energy bright excited state is also shown with an open symbol. All results obtained with def2-SVP. To aid visualisation, the results are divided into coloured sections based on the number of silicon atoms in the nanoparticle (blue, yellow, green and grey for 10, 35, 84 and 165 silicon atoms, respectively).

Fig. 5 shows how the exciton binding energy, the difference between the fundamental and optical gap, of the SiNPs changes when replacing hydrogen capping atoms with methyl groups and/or increasing the size of the silicon core. The trend in exciton binding energy mirrors that of the fundamental and optical gap, discussed above, increasing as the size of the silicon core decreases. Similar, as observed in our previous work, while evGW-BSE and TD-DFT predict very similar trends, the absolute values are rather different, with the TD-DFT values being significantly smaller. The latter is likely caused by an accumulation of errors, arising from the fact that the fundamental gap, as approximated by the Kohn–Sham gap, is not necessarily internally consistent with the optical gap from TD-DFT. Overall, both evGW-BSE and TD-DFT predict that the excitation corresponding to the optical gap and other low-energy excitations for the methyl-terminated SiNPs are, just as those of their hydrogen-terminated counterparts, clearly excitonic in nature.

The evGW-BSE predicted exciton binding energy of Si_84_H_12_(CH_3_)_52_ and the TD-DFT predicted exciton binding energy of Si_35_H_12_(CH_3_)_24_ are slightly higher than expected from their respective trends. The origin for this, at least in the case Si_84_H_12_(CH_3_)_52_ is that this particle has a slightly larger fundamental gap than Si_84_H_52_(CH_3_)_12_ but a smaller optical gap and that the exciton binding energy being a difference magnifies this.

### (De)localisation

**Fig. 6:**
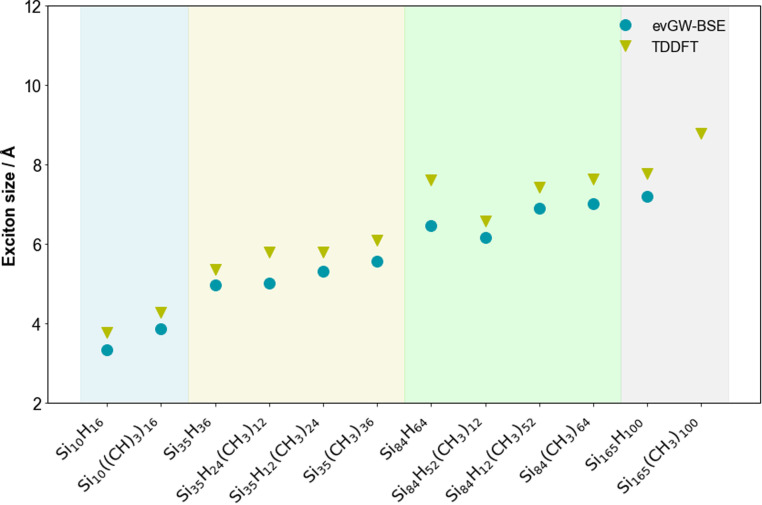
Predicted exciton size of the lowest energy exciton for the hydrogen and methyl-terminated SiNPs. All values calculated with def2-SVP. To aid visualisation, the results are divided into coloured sections based on the number of silicon atoms in the nanoparticle (blue, yellow, green and grey for 10, 35, 84 and 165 silicon atoms, respectively).

Fig. 6 shows how the exciton radius, defined as the root-mean-square separation of the electron and hole component of the exciton, of the SiNPs changes when replacing hydrogen capping atoms with methyl groups and/or increasing the size of the silicon core. Comparing SiNPs with similar degrees of methylation then just as for hydrogen-terminated SiNPs the exciton radius increases with increasing size of the silicon core. Additionally, the fully methyl-terminated particles always have a larger exciton radius than their hydrogen-terminated counterparts, even if for Si_84_ the difference is small.

**Fig. 7:**
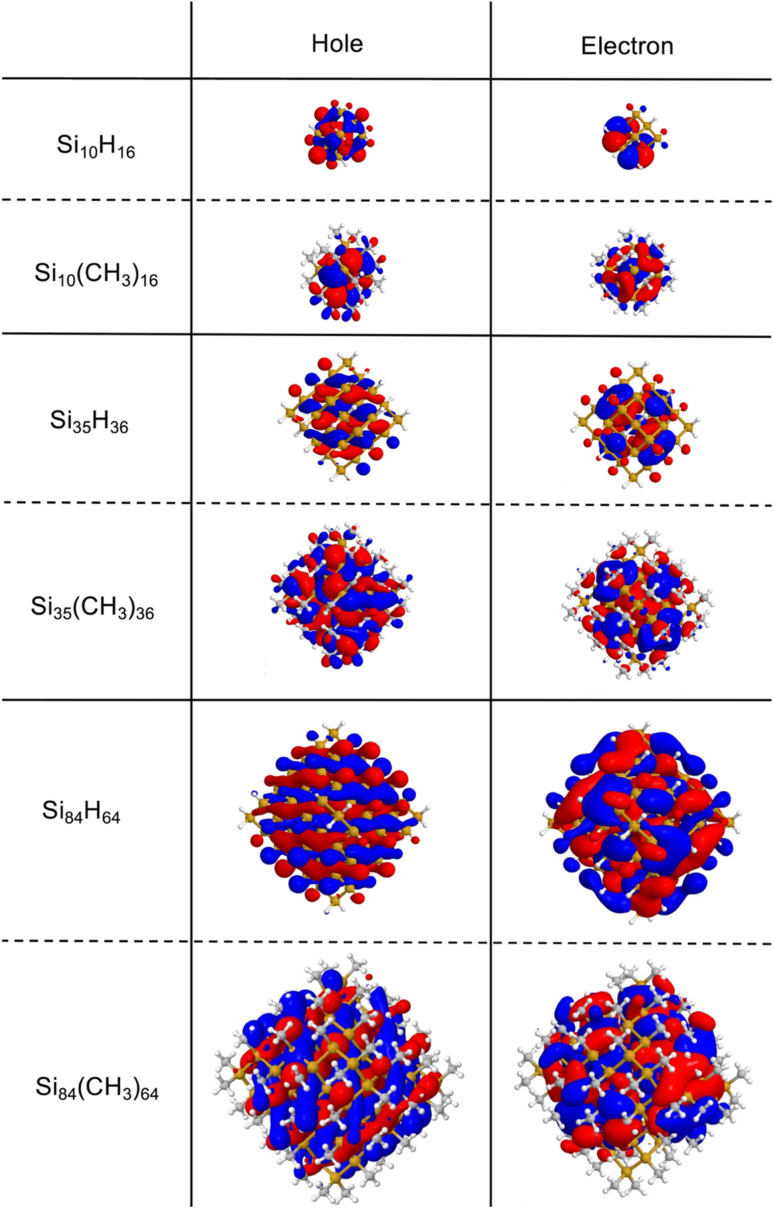
Leading natural transition orbitals (NTOs) for the pristine and fully-methyl-terminated SiNPs calculated *via* evGW-BSE, B3LYP, def2-SVP using *C*_1_ symmetry. Because the calculations were performed using the *C*_1_ symmetry group for the hydrogen-terminated nanoparticles, instead of the *T*_d_ point group, the triply degenerate *T*_2_ excited state is represented as three separate degenerate *C*_1_ excited states. For each hydrogen-terminated particle, only the NTOs of one of these degenerate states are shown here. See Fig. S1 and S2† for the corresponding figures for partially methyl substituted particles.

Fig. 7 shows the leading natural transition orbitals for the hole and excited electron component of the lowest excited state corresponding to the optical gap of the fully hydrogen and fully methyl-terminated SiNPs. As expected from the exciton size data discussed above, these natural transition orbitals are generally well delocalised over the volume of the particles for both the hydrogen and methyl-terminated particles.

**Fig. 8:**
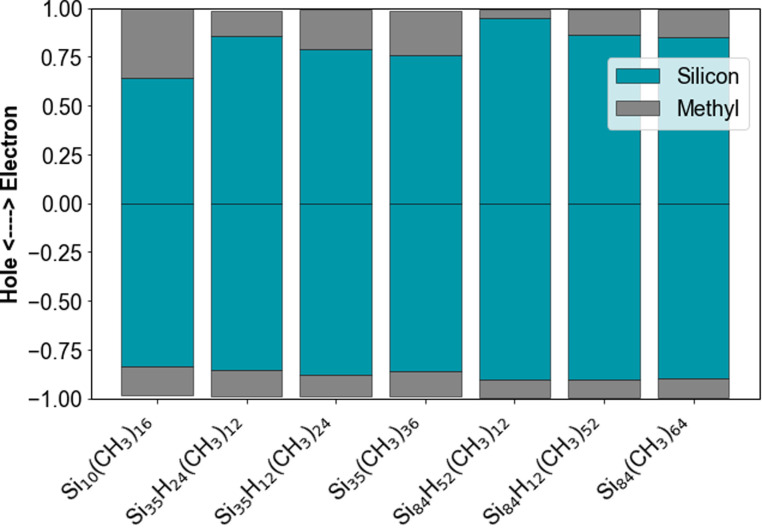
Contribution of the silicon core and methyl ligands to the hole and electron component of the lowest exciton of the methyl-terminated SiNPs. Results obtained from a TheoDORE analysis of the lowest excited states in evGW-BSE calculations on top of DFT calculations with the B3LYP functional and def2-SVP basis set. See Fig. S3† for the TD-DFT equivalent.

The natural transition orbitals in Fig. 7 also show clear evidence of delocalisation of the excited state not only over the silicon core, but also, depending on the specific SiNP, a significant fraction of the methyl groups. This involvement of the methyl groups is also apparent from Fig. 8 which shows the contribution of the methyl groups and the silicon core to the hole and excited electron component of the lowest excited state.

Based on Fig. 8 (and its TD-DFT equivalent S7†), typically 8–14% of the hole is localised on the methyl groups and 10–36% of the excited electron. The contribution of the methyl groups to the excited electron increases with increasing replacement of hydrogen capping atoms by methyl groups. Similarly, the contribution of the methyl groups to the hole decreases with increasing replacement of hydrogen capping atoms by methyl groups. The contribution of the methyl groups to the excited electron and hole also appears to decrease with the size of the silicon core and hence the capping ligands to silicon atoms in the core ratio.

### (De)localisation in reciprocal space

**Fig. 9:**
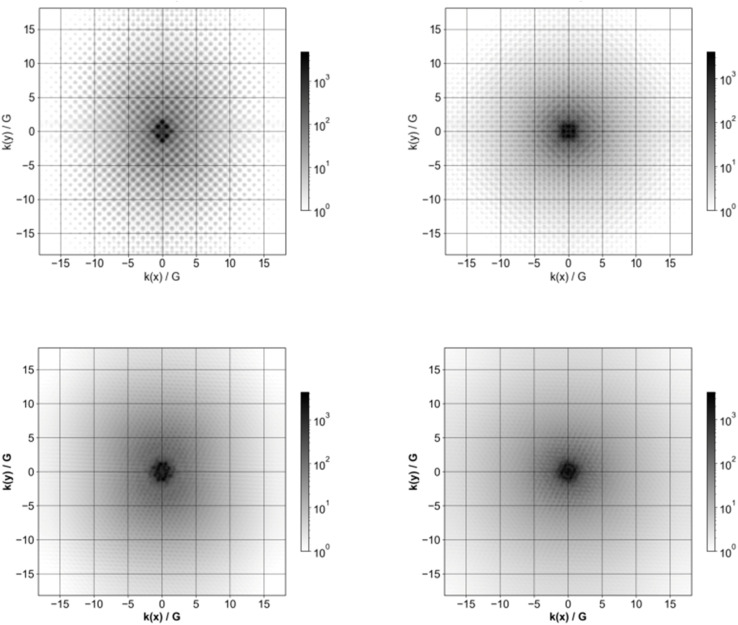
Fourier transform of the highest occupied quasiparticle state (left) and lowest unoccupied quasiparticle state (right) for Si_84_H_64_ (top row) and Si_84_(CH_3_)_64_ (bottom row). In the case of Si_84_H_64_ the highest occupied quasiparticle state is triply degenerate and the Fourier transform of only one of the three is shown.

Fig. 9 shows Fourier transforms of the orbitals corresponding to the highest occupied and lowest unoccupied quasiparticle state of Si_84_H_64_ and Si_84_(CH_3_)_64_. In our previous study on hydrogen-terminated silicon particles, we demonstrated that with increasing nanoparticle size, the Fourier transform of the frontier molecular orbitals and thus the frontier quasiparticle states and hence their delocalisation in reciprocal space, become less diffuse and sharper. Here, we consider the impact of methyl ligands on the delocalisation of the frontier quasiparticle states in reciprocal space. The comparison between the Fourier transforms for the pristine Si_84_H_64_ and Si_84_(CH_3_)_64_ nanoparticles in Fig. 9 shows that the latter are more diffuse and less sharp than the former. The frontier orbitals and hence quasiparticle states thus become more smeared out in reciprocal space when replacing hydrogen atoms by methyl groups. Moreover, as can be seen from Fig. S8† there is still a variation in the extent of how smeared out the frontier molecular orbital are in reciprocal space as function of the size of the methyl-terminated nanoparticles; the Si_10_(CH_3_)_16_ orbitals are still the most smeared out in line with the fact that it’s the smallest nanoparticle.

### Spectra

**Fig. 10:**
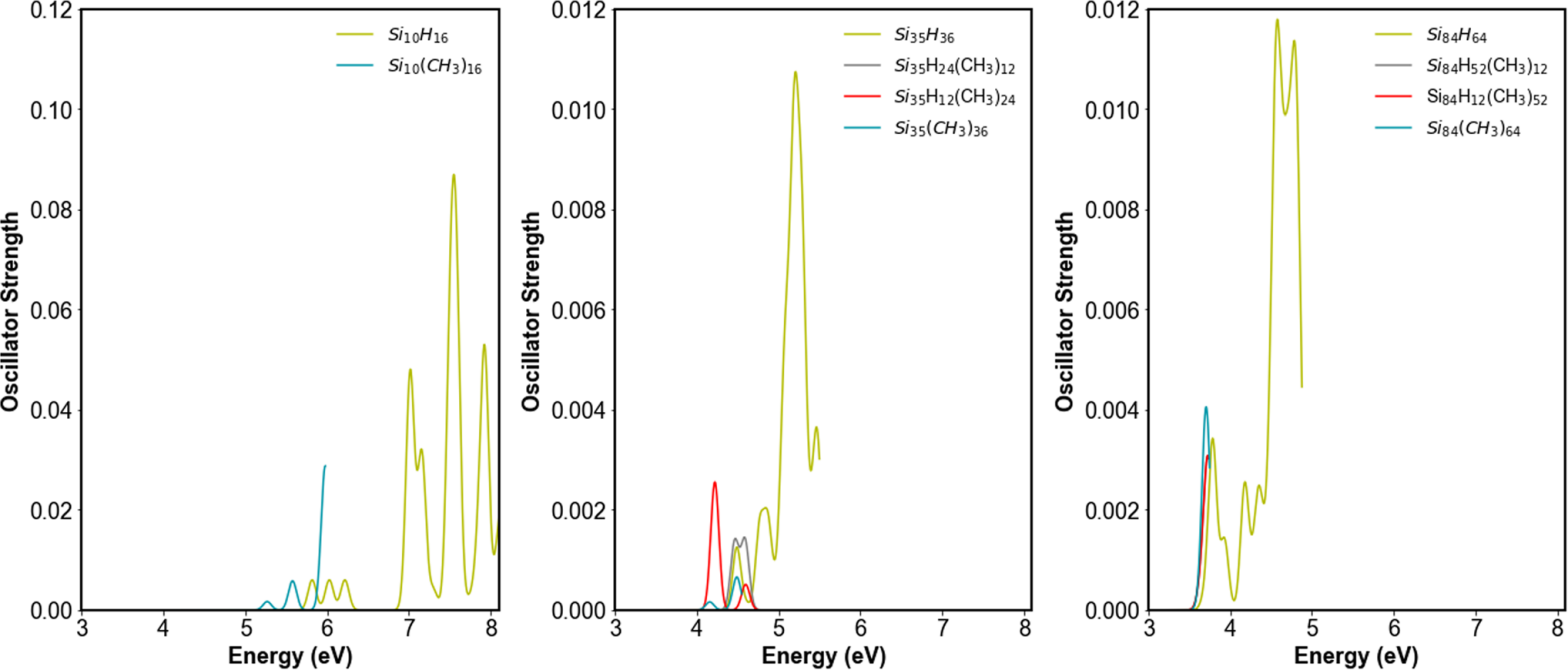
Absorption spectra of the hydrogen-terminated SiNPs *versus* their partially methyl-terminated and fully methyl-terminated counterparts as predicted using TD-DFT, *C*_1_ symmetry, the B3LYP functional, def2-SVP basis set and a Gaussian broadening of 0.05 eV.

Fig. 10 shows the TD-DFT predicted spectra of the hydrogen and (partially) methyl terminated particles approximated by Gaussian functions centred at the vertical excitation energies with heights proportional to the excitations' oscillator strengths (*i.e.* neglecting vibronic effects, which would be computationally intractable to include for particles of this size). The spectra of the fully methyl-terminated nanoparticles exhibit a red shift compared to their hydrogen-terminated counterparts, in line with the reduction of the optical gap discussed above. There is an observable overlap in absorption at lower energies between both types of nanoparticles. Also, the spectra of the fully methyl-terminated nanoparticles show similar oscillator strengths as their hydrogen-terminated counterparts, implying a similar absorption of light. Like the hydrogen-terminated nanoparticles, the trend of a red-shift in the spectra with increasing particle size persists for the methyl-terminated nanoparticle.

In previous experimental studies, it was observed that when going from hydrogen to alkyl terminated SiNPs, the exciton lifetime decreases and the radiative rate increases.^24^ Subsequent tight-binding and DFT calculations discussed in the introduction also predicted that switching hydrogen atoms for alkyl groups reduces exciton lifetimes and improves radiative rates.^24,25^ We observe that for all (partially) methyl-terminated particles studied, the oscillator strength of the lowest bright excited state increases relative to the corresponding hydrogen-terminated SiNPs, again neglecting vibronic effects. Such an increase in oscillator strength would fit with the experimentally observed reduced lifetimes and improved radiative rates, if it was not for the fact that for the fully methyl-terminated SiNPs and SiNPs terminated with methyl groups on only the faces there is always, as discussed above, a non-bright excited-state lying slightly lower in energy. Moreover, while the oscillator strength increases when replacing hydrogen atoms by methyl groups, the optical gap also decreases, as discussed above, and as the radiative rate is proportional to the optical gap squared, this decrease in the optical gap might more than compensate for the increased oscillator strength. As exciton lifetimes and radiative rates are inherently measured by emission, it is possible that excited state relaxation results in the bright state becoming the lowest state before emission. This might be more likely to be the case for larger particles as the gap between the lowest non-bright and bright states deceases with increasing particle size and the states end up effectively degenerate for larger particles. The difference in the optical gap when replacing hydrogen atoms by methyl groups also gets smaller with increasing particle size meaning that effect of the introduction of methyl groups on the radiative rate and lifetime is more likely to be dominated by the change in oscillator strength for these larger particles. As the oscillator strength values for both hydrogen and (partially) methyl-terminated SiNPs are small, finally, vibronic effects on the predicted absolute values of the oscillator strengths can be significant. However, there's no *a priori* reason to believe that these effects will be more significant for either hydrogen or (partially) methyl terminated SiNPs and hence change the relative oscillator strength values of SiNPs with different surface terminations.

## Discussion

From the results above it is clear that changing the termination of the surface of SiNPs, and hence the nature of their passivation, influences the electronic and optical properties of the nanoparticles. The methyl group firstly lower the symmetry of the particles. However, the symmetry of the silicon core of the particles stays approximately tetrahedral. The splitting of what would be triply degenerate excited states for the hydrogen-terminated particles, is typically less than 0.005 eV for the fully methyl-terminated particles. That said, the disorder of the methyl group clearly results in a smearing out of the frontier orbitals of the methyl-terminated particles in reciprocal space and in that sense, makes these particles less ‘bulk like’, less ‘crystalline’, than their hydrogen-terminated counterparts.

Beyond geometrical changes, it is tempting to explain the reduction of the optical and fundamental gaps of the methyl-terminated particles relative to their hydrogen-terminated particles in terms of the fact that methyl groups are a stronger electron donor than hydrogen atoms. This explanation would be in line with the observation that the ionisation potential and electron affinity both shift up to less negative, shallower, values when gradually replacing hydrogen atoms by methyl groups, as well as the fact that generally, the shift for the ionisation potential is larger than that for the electron affinity.

The electron donating property of the methyl group, however, cannot be the whole story. Both the change in exciton size, implicitly, and the analysis of the character of the natural transition orbitals, explicitly, show that the excitons delocalise not only over the silicon core, but also partially over the terminating methyl groups. The methyl capping groups hence are not only not innocent, in the sense that their presence changes the particles properties, but this non-innocence also is not simply the effect of the capping groups being an external perturbation to the particles. The methyl groups clearly partake in the excitons, something that cannot be explained in terms of their electron donating ability, which is a ground state property.

Based on the contribution of the methyl groups to the natural transition orbitals, as shown in Fig. 8, both the hole and excited electron partly delocalise over the methyl groups. Even if more of the excited electron than the hole delocalises, it is clear that these excitons are not charge-transfer excitons. As such, the underlying occupied and unoccupied methyl orbitals must have a similar energy as those for the silicon core so that the exciton can spill over onto the methyl groups. The increase in the size of the exciton resulting from the spill-over then should reduce the exciton binding energy when replacing hydrogen atom by methyl groups, which indeed can be seen in Fig. 5, and the change in the optical gap should be smaller than that in the fundamental gap, which indeed generally seems to be the case. In summary, the reduction in fundamental gap is mostly likely due to just the electron donating effect of the methyl groups. However, to explain why the optical gap of the methyl-terminated particles is smaller than their hydrogen-terminated counterparts and why this change in the optical gap is smaller than that for the fundamental gap requires a combination of the electron donating effect of the methyl groups and the spill-over of the exciton on the methyl groups.

Finally, the effect of replacing hydrogen atoms with methyl groups is largest for the smallest particle. Both the shift in the fundamental and optical gap of the methyl-terminated particles relative to their hydrogen capped counterparts and the relative contribution of the methyl groups to the natural transition orbitals decreases with increasing size of the silicon core. This is most likely simply the result of the decreasing surface to volume ratio with increasing core size and the fact that there is thus increasingly more silicon core relative the methyl capping groups. The decreasing effect and contribution of the methyl groups with increasing particle size also means that hydrogen-terminated silicon nanoparticles become increasingly good models of methyl-terminated silicon nanoparticles with increasing particle size. Moreover, while we haven't explicitly studied termination with larger alkyl groups here, the effect is probably similar for them, other than steric effects which will increase with increasing alkyl chain lengths.

## Conclusions

Switching from hydrogen to methyl surface termination of SiNPs is found to result in a reduction of the fundamental gap, optical gap and exciton binding energy of the particles. This effect of changing the capping groups becomes smaller with increasing size of the silicon core, which is probably simple the result of the decreasing surface to volume ratio. As a result, hydrogen-terminated SiNPs become increasingly good models of methyl-terminated and more generally alkyl-terminated silicon nanoparticles with increasing particle size.

The exciton-size increases when replacing hydrogen atoms with methyl groups for the same silicon core, which is suggestive of the exciton spilling over from the silicon core onto the methyl groups. This interpretation is supported by an analysis of the character of the leading natural transition orbital for the lowest energy exciton for the different particles which shows that that the methyl groups contribute to both the excited electron and hole component of the exciton. While the contribution to the excited electron component is larger than to that for the hole, it is also clear that these lowest energy excitons do not correspond to charge-transfer states. We finally propose that the smaller fundamental gap, optical gap and exciton binding energy values of the methyl-terminated particles relative to their hydrogen-terminated counterparts is due to a combination of the electron donating nature of methyl groups and the spill-over of the exciton on the methyl groups.

## Conflicts of interest

There are no conflicts of interest to declare.

## Supplementary Material

RA-015-D5RA03272E-s001

RA-015-D5RA03272E-s002

## Data Availability

The data supporting this article have been included as part of the ESI.†
