# Supplementary material for: The effect of alkyl termination on the optical and electronic properties of silicon nanoparticles
Source: RSC Adv. 2025 Jun 17;15(26):20453–63. doi: 10.1039/d5ra03272e (PMC12172018; doi:10.1039/d5ra03272e)
Supplement: RA-015-D5RA03272E-s001 [file RA-015-D5RA03272E-s001.pdf]

Supplementary Information (SI) for RSC Advances.
This journal is © The Royal Society of Chemistry 2025

Supporting Information: The Effect of Alkyl Termination on the Optical and Electronic Properties of Silicon Nanoparticles

Eimear Madden, Martijn A. Zwijnenburg*

Department of Chemistry, University College London, 20 Gordon Street, London WC1H 0AJ, U.K.
m.zwijnenburg@ucl.ac.uk

Table S1: *Kohn-Sham (KS) highest-occupied molecular orbital (HOMO) and lowest-unoccupied molecular orbital (LUMO) and the KS HOMO-LUMO gap values as calculated using DFT and highest occupied (–IP) and lowest unoccupied (–EA) quasiparticle states and the fundamental gap* (D_f) *as calculated using evGW-BSE. All results obtained using the def2-SVP basis-set. All values in eV.*

	DFT			evGW		
**Nanoparticle**	**KS-HOMO**	**KS-LUMO**	**KS-Gap**	**-IP**	**-EA**	**Gap**
$Si_{10}H_{16}$	-7.54	-1.00	6.54	-9.21	0.91	10.12
$Si_{10}(CH_3)_{16}$	-6.1	-0.42	5.68	-7.44	1.28	8.72
$Si_{35}H_{36}$	-6.73	-1.63	5.1	-7.77	-0.17	7.60
$Si_{35}H_{24}(CH_3)_{12}$	-6.28	-1.24	5.04	-7.30	0.11	7.41
$Si_{35}H_{12}(CH_3)_{24}$	-5.95	-1.22	4.73	-6.88	0.07	6.95
$Si_{35}(CH_3)_{36}$	-5.64	-1.24	4.40	-6.50	0.26	6.76
$Si_{84}H_{64}$	-6.29	-2.07	4.22	-6.94	-0.97	5.97
$Si_{84}H_{52}(CH_3)_{12}$	-6.03	-1.81	4.22	-6.70	-0.73	5.97
$Si_{84}H_{12}(CH_3)_{52}$	-5.42	-1.35	4.06	-6.402	-0.35	6.05
$Si_{84}(CH_3)_{64}$	-5.22	-1.20	4.02	-5.85	-0.21	5.64
$Si_{165}H_{100}$	-6.00	-2.49	3.51	-6.36	-1.67	4.69
$Si_{165}(CH_3)_{100}$	-4.98	-1.42	3.56			

Table S2: *Energies of the highest occupied (–IP) and lowest unoccupied (–EA) quasiparticle states and fundamental gap* (D_f) *values as calculated using qsGW in combination with the def2-SVP basis set. All values in eV.*

**Simulation details**	**qsGW**		
**Nanoparticle**	**-IP**	**-EA**	D_f
$Si_{10}H_{16}$	-9.42	0.69	10.11
$Si_{10}(CH_3)_{16}$	-7.85	1.03	8.88
$Si_{35}H_{36}$	-8.08	-0.49	7.59

Table S3: *Kohn-Sham (KS) highest-occupied molecular orbital (HOMO) and lowest-unoccupied molecular orbital (LUMO) and the KS HOMO-LUMO gap values as calculated using DFT and highest occupied (–IP) and lowest unoccupied (–EA) quasiparticle states and the fundamental gap (*$\mathbf{D}_f$*) as calculated using evGW-BSE. All results obtained using the def2-TZVP basis-set. All values in eV.*

	**DFT**			**evGW**		
**Nanoparticle**	**KS-HOMO**	**KS-LUMO**	**KS-Gap**	**-IP**	**-EA**	**Gap**
$Si_{10}H_{16}$	-7.54	-1.16	6.32	-9.29	0.33	9.62
$Si_{10}(CH_3)_{16}$	-6.14	-0.73	5.41	-7.76	0.49	8.25
$Si_{35}H_{36}$	-6.73	-1.63	5.1	-7.74	-0.23	7.51
$Si_{35}H_{24}(CH_3)_{12}$	-6.29	-1.37	4.91			
$Si_{35}C_{24}H_{84}$	-5.99	-1.43	4.56			
$Si_{35}(CH_3)_{36}$	-5.63	-1.24	4.40			
$Si_{84}H_{64}$	-6.29	-2.10	4.19			

Table S4: *Optical gap values (Do) as calculated using TDDFT, evGW-BSE, in combination with the def2-SVP basis set. All values in eV. For $Si_{10}H_{16}$, for which the lowest excited state is not optically allowed, and for $Si_{10}(CH_3)_{15}$, for which the lowest excited-state is clearly dark, the gap towards the lowest bright excited state is given in between parentheses.*

	**TDDFT**	**evGW-BSE**	**qsGW-BSE**
**Nanoparticle**	**Δ_o**	**Δ_o**	**Do**
$Si_{10}H_{16}$	5.72 (5.82)	6.19 (6.37)	6.17 (6.43)
$Si_{10}(CH_3)_{16}$	4.94(5.28)	5.34(5.70)	5.43 (5.84)
$Si_{35}H_{36}$	4.47	4.82	4.85
$Si_{35}H_{24}(CH_3)_{12}$	4.45 (4.46)	4.82	
$Si_{35}C_{24}H_{84}$	4.15 (4.22)	4.46 (4.54)	
$Si_{35}(CH_3)_{36}$	4.02 (4.17)	4.37 (4.51)	
$Si_{84}H_{64}$	3.69	3.87	
$Si_{84}H_{54}(CH_3)_{12}$	3.68(3.69)	3.87 (3.88)	
$Si_{84}H_{12}(CH_3)_{52}$	3.59 (3.65)	3.78(3.85)	
$Si_{84}(CH_3)_{64}$	3.55 (3.63)	3.77 (3.85)	
$Si_{165}H_{100}$	3.15	3.22	
$Si_{165}(CH_3)_{100}$	3.15	-	

Table S5: *Optical gap values (Do) as calculated using TDDFT, evGW-BSE, in combination with the def2-TZVP basis set. All values in eV. For $Si_{10}H_{16}$, for which the lowest excited state is not optically allowed, and for $Si_{10}(CH_3)_{15}$, for which the lowest excited-state is clearly dark, the gap towards the lowest bright excited state is given in between parentheses.*

	**TDDFT**	**evGW-BSE**
**Nanoparticle**	**Δ_o**	**Δ_o**
$Si_{10}H_{16}$	5.52 (5.79)	5.67 (5.92)
$Si_{10}(CH_3)_{16}$	4.67 (5.04)	4.96 (5.35)
$Si_{35}H_{36}$	4.47	4.55
$Si_{35}H_{24}(CH_3)_{12}$	4.32(4.35)	
$Si_{35}C_{24}H_{84}$	3.97(4.08)	
$Si_{35}(CH_3)_{36}$	3.83(3.91)	
$Si_{84}H_{64}$	3.59	

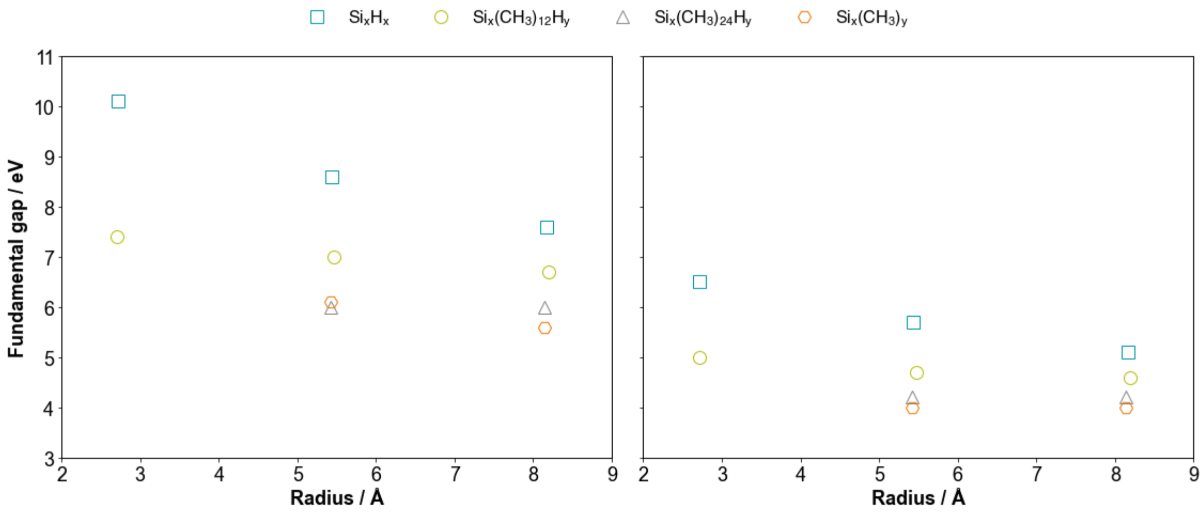

Fig. S1 *Fundamental gap versus particle size as calculated via evGW (left) and DFT (right) in combination with the def2-SVP basis-set for the hydrogen and methyl-terminated silicon nanoparticles.*

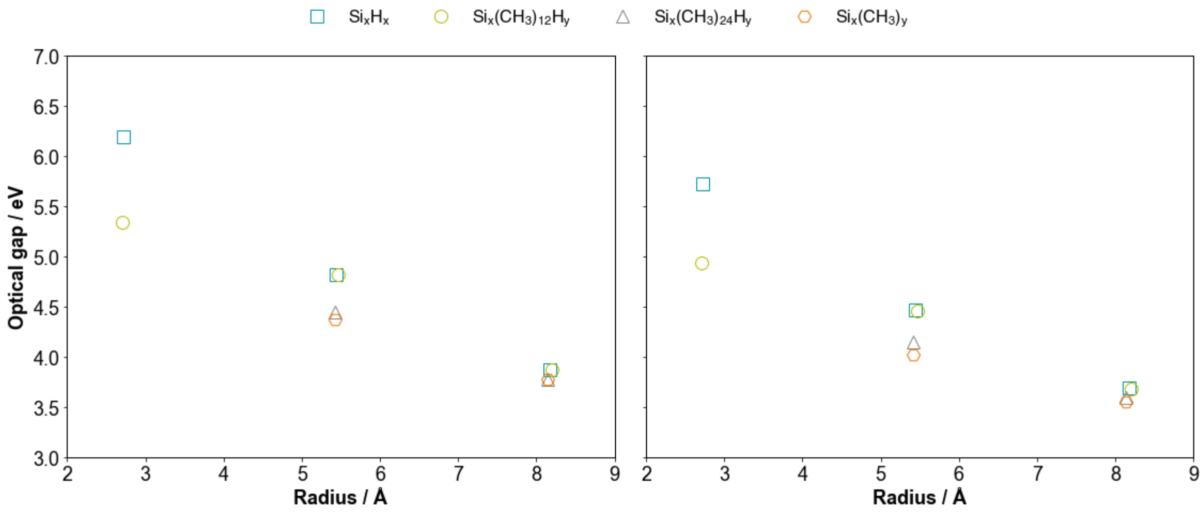

Fig. S2 *Optical gap versus particle size as calculated via evGW-BSE (left) and TDDFT (right) in combination with the def2-SVP basis-set for the hydrogen and methyl-terminated nanoparticles.*

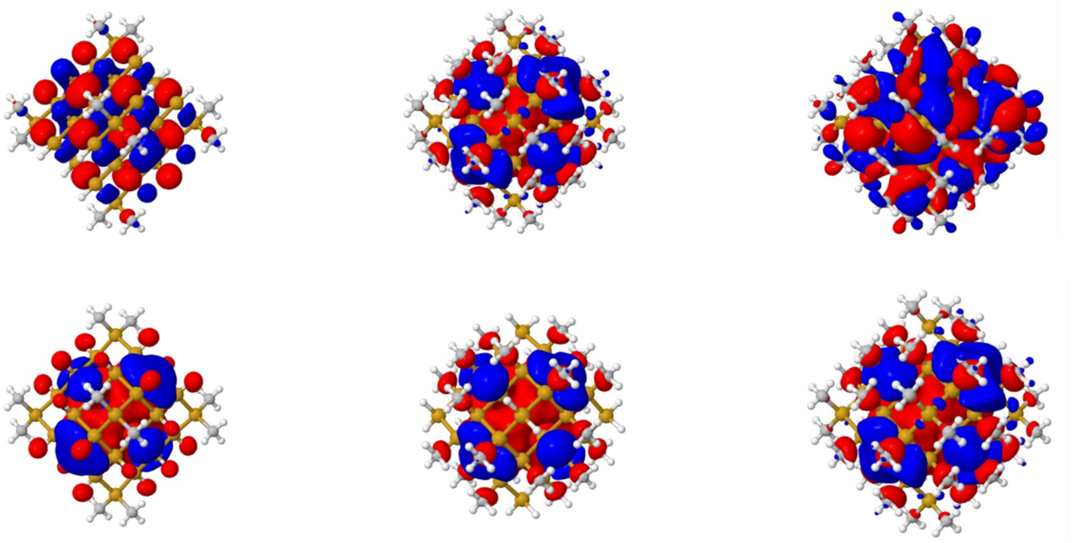

Fig. S3 *Leading NTOs for Si35-nanoparticle with varying degree of methyl substitution. Top; hole orbital. Bottom; electron orbital. From left to right:* $Si_{35}H_{24}(CH_3)_{12}$, $Si_{35}H_{12}(CH_3)_{24}$, $Si_{35}(CH_3)_{36}$.

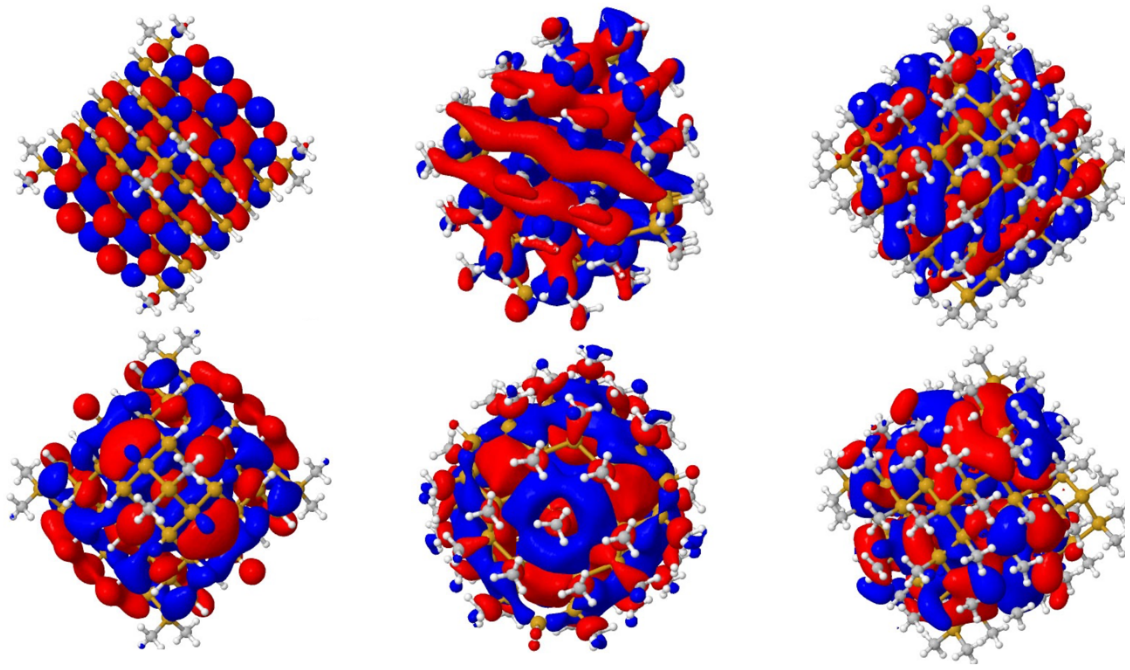

Fig. S4 *Leading NTOs for Si84-nanoparticle with varying degree of methyl substitution. Top; hole orbital. Bottom; electron orbital. From left to right:* $Si_{84}H_{52}(CH_3)_{12}$, $Si_{84}H_{12}(CH_3)_{52}$, $Si_{84}(CH_3)_{64}$.

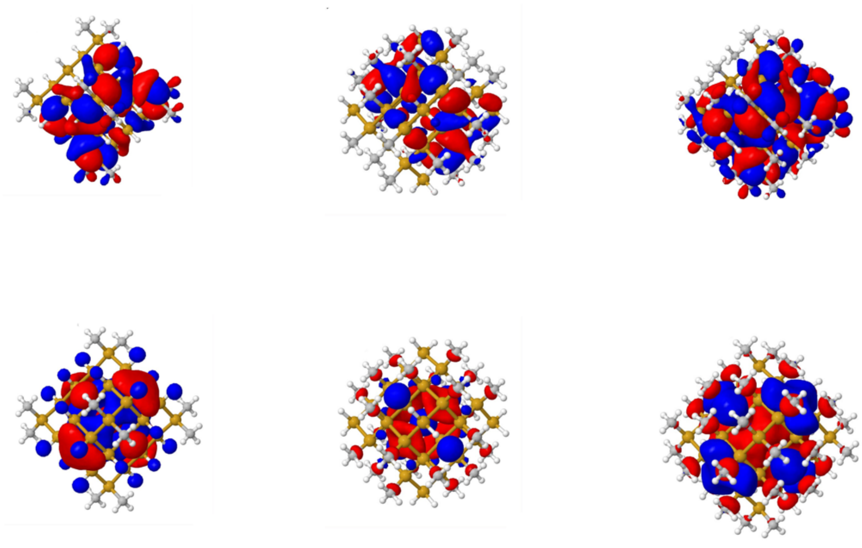

Fig. S5 *Highest occupied and lowest unoccupied orbitals for the Si35-nanoparticle with varying degree of methyl substitution. From left to right:* $Si_{35}H_{24}(CH_3)_{12}$, $Si_{35}H_{12}(CH_3)_{24}$, $Si_{35}(CH_3)_{36}$.

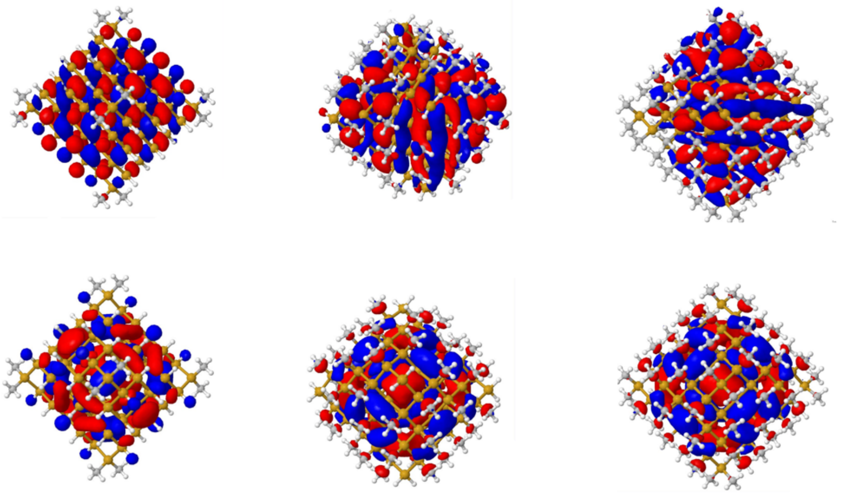

Fig. S6 *Highest occupied and lowest unoccupied orbitals for for Si84-nanoparticle with varying degree of methyl substitution. From left to right:* $Si_{84}H_{52}(CH_3)_{12}$, $Si_{84}H_{12}(CH_3)_{52}$, $Si_{84}(CH_3)_{64}$.

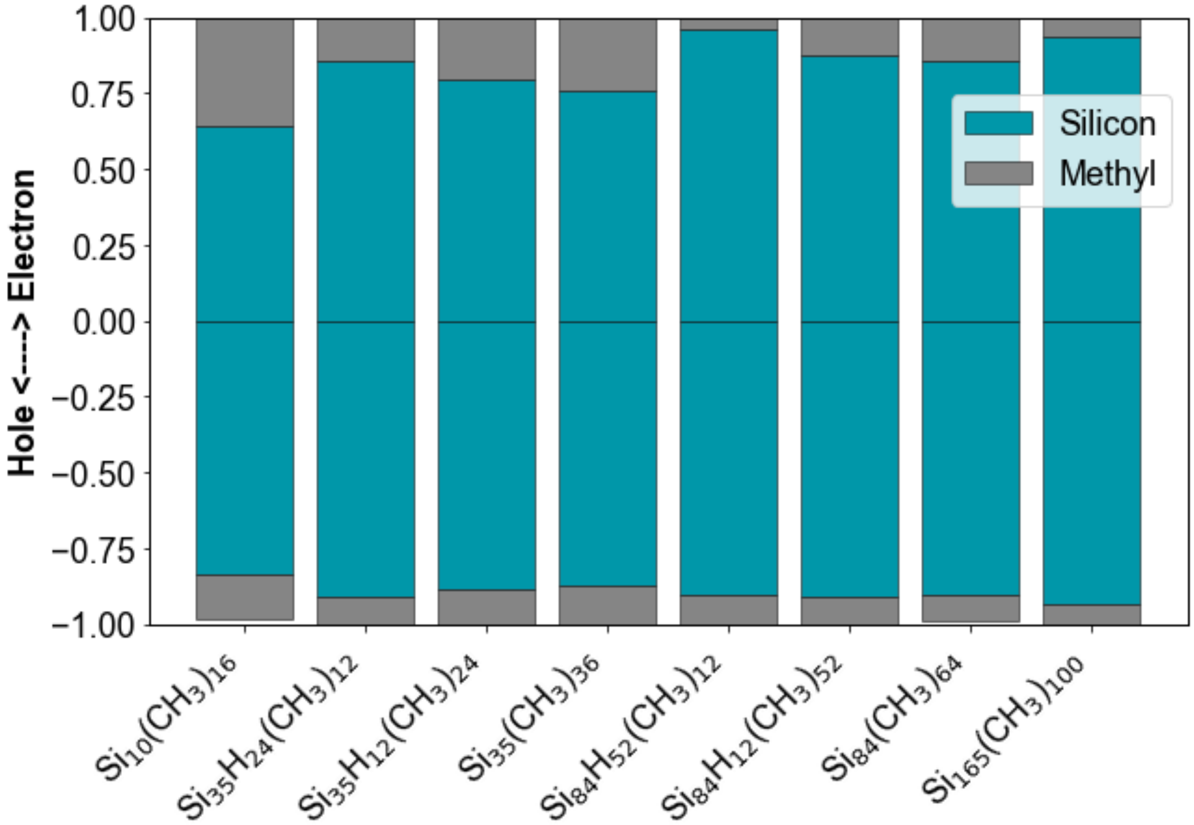

Fig. S7 *Contribution of the silicon core and methyl ligands to the hole and electron component of the lowest exciton of the methyl-terminated SiNPs obtained via TheoDORE analysis from TDDFT calculations, B3LYP and def2-SVP basis set*

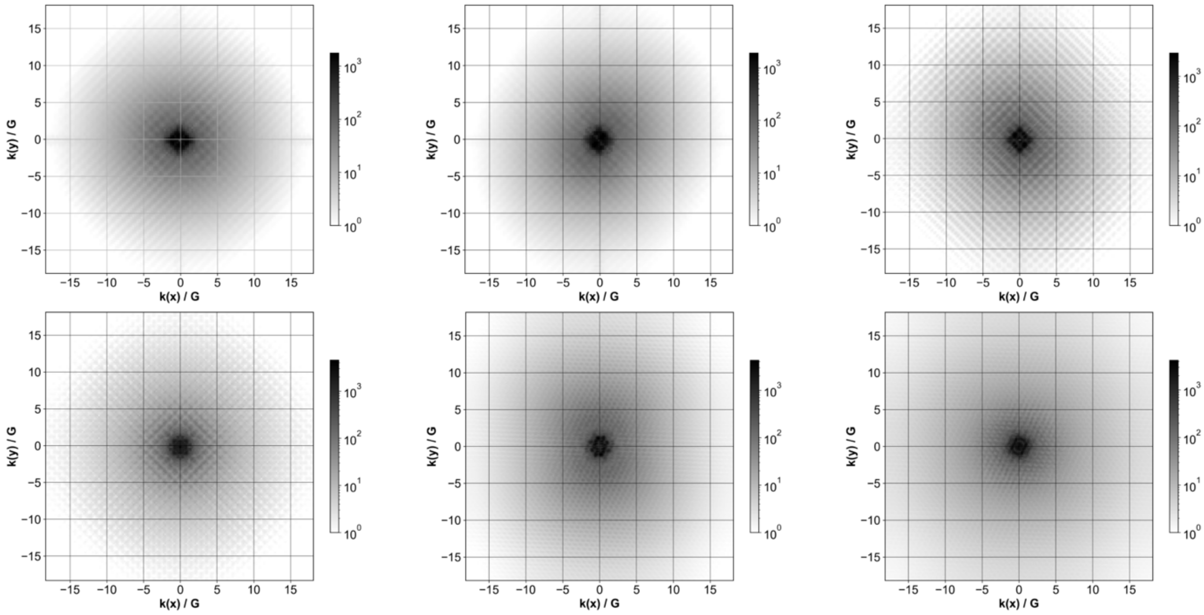

Fig. S8 *Fourier transform of HOMO (top) and LUMO (bottom) for methyl-terminated SiNPs with no hydrogen termination. From left to right:* $Si_{10}(CH_3)_{16}$, $Si_{35}(CH_3)_{36}$, $Si_{84}(CH_3)_{64}$.

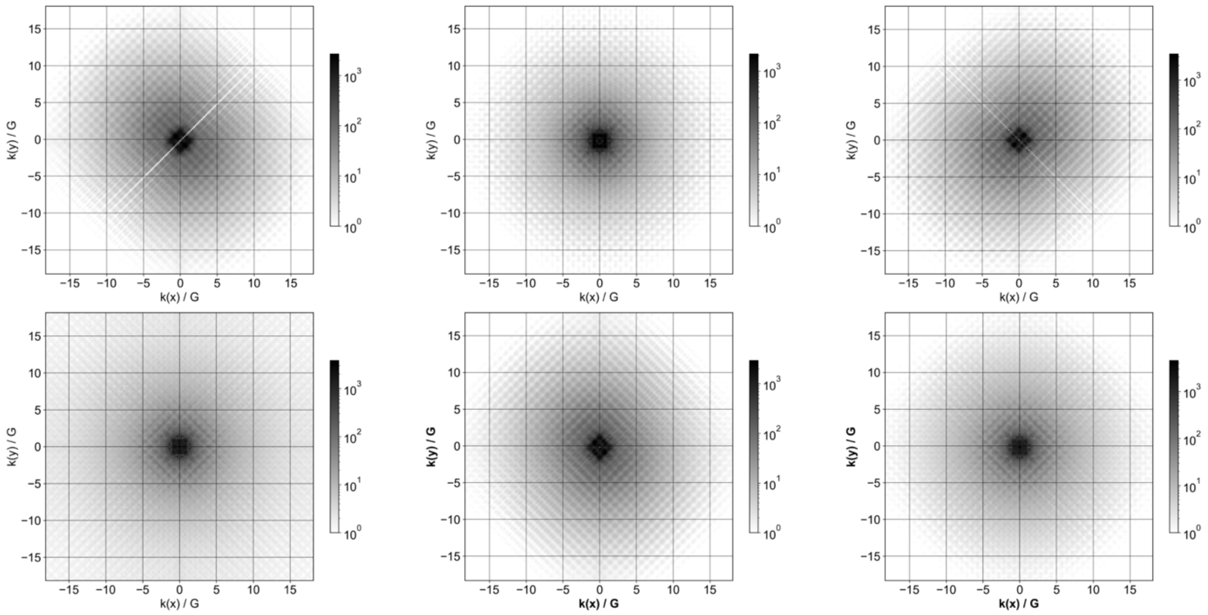

Fig. S9 *Fourier transform of HOMO (top) and LUMO (bottom) for Si35-nanoparticle with varying degree of methyl substitution. From left to right:* $Si_{35}H_{24}(CH_3)_{12}$, $Si_{35}H_{12}(CH_3)_{24}$, $Si_{35}(CH_3)_{36}$.

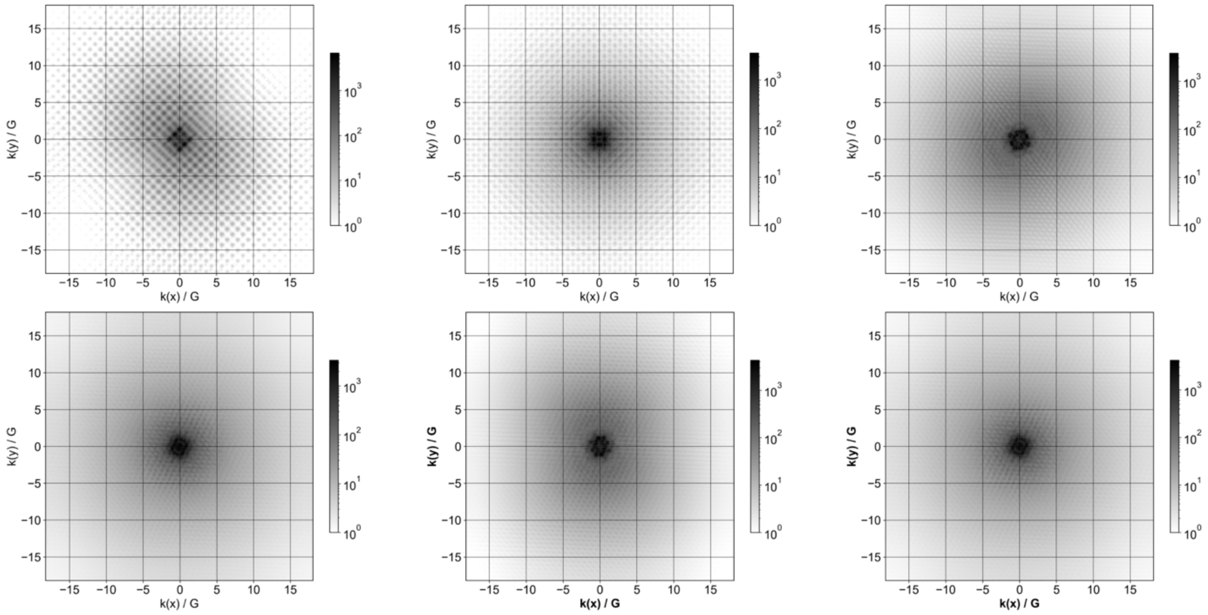

Fig. S10 *Fourier transform of HOMO (top) and LUMO (bottom) for Si84-nanoparticle with varying degree of methyl substitution. From left to right:* $Si_{84}H_{52}(CH_3)_{12}$, $Si_{84}H_{12}(CH_3)_{52}$, $Si_{84}(CH_3)_{64}$.
